# Segmental Testicular Infarction in the Setting of Heroin Use

**DOI:** 10.7759/cureus.110989

**Published:** 2026-06-16

**Authors:** Jeremiah Hutson, Obadiah Kirk, Muhammad Nabeel, Claire Mutch, Rohan Kumar

**Affiliations:** 1 Medical Education, Michigan State University College of Human Medicine, Grand Rapids, USA; 2 Medical Education, Michigan State University College of Human Medicine, Flint, USA; 3 Internal Medicine, University of Michigan Health - Sparrow Hospital, Lansing, USA; 4 Neurology and Opthalmology, University of Michigan Health - Sparrow Hospital, Lansing, USA

**Keywords:** hypercoagulability, polysubstance use, segmental testicular infarction, vasoconstriction, vasoconstrictive

## Abstract

Segmental testicular infarction (STI) is a rarely documented cause of acute scrotal pain likely resulting from localized ischemia of testicular tissue. It is most often commonly associated with torsion, infection, trauma, or hypercoagulable states, though idiopathic cases can occur. Substance use has been sparsely reported as a potential contributing factor. In this case report, we present a case of STI in a patient with recent heroin use, highlighting a possible association and emphasizing the importance of imaging in acute testicular pain. A 48-year-old male with a history of polysubstance use presented with progressively worsening right testicular pain. He reported heroin use four days prior and described a history of intermittent, self-resolving testicular pain over several years. Physical examination revealed a heterogeneous right testicle with focal firmness. Laboratory evaluation, including tumor markers, was unremarkable. Doppler ultrasound demonstrated heterogeneous echotexture and reduced vascular flow in the right testicle. MRI of the pelvis showed hypoenhancement and absent diffusion signal consistent with segmental infarction. The patient was managed conservatively with analgesia and instructions for outpatient urology follow-up. STI may present with variable and chronic symptoms, making diagnosis difficult. This case illustrates heroin use as a possible contributing factor, possibly through vasoconstriction or prothrombotic effects, leading to compromised testicular perfusion. Clinicians should consider STI in patients with atypical or chronic testicular pain, particularly in the setting of substance use. Early imaging is critical for guiding management and avoiding unnecessary surgical intervention.

## Introduction

Segmental testicular infarction (STI) is a rare condition, with fewer than 200 cases described in the literature, characterized by localized necrosis of a portion of the testicle due to compromised blood flow [1,2]. It is a condition generally caused by testicular torsion, epididymitis, trauma, and systemic factors such as hypercoagulability; etiology remains open, with varying clinical pictures [3]. Commonly, patients present with acute testicular pain, tenderness, neoplasms, and swelling [4-6]. Doppler ultrasound is crucial for STI diagnosis, as it detects areas of reduced blood flow. Treatment may range from conservative management for small infarctions to surgical intervention, including orchiectomy, for more severe cases. Early recognition and timely intervention are crucial for increasing the feasibility of conservative management, preventing testicular atrophy while improving prognosis, avoiding unnecessary orchiectomy, and preserving fertility [7-9].

## Case presentation

The subject is a 48-year-old male with a past medical history of polysubstance abuse. He presented to the emergency department due to severe right testicular pain. He also admitted to using heroin approximately four days prior. The patient stated that approximately five years ago, he had developed severe testicular pain after injecting heroin, and his right testicle had significantly grown in size. At that time, he had taken several other drugs, and within two days, his testicle had returned to normal size. Since then, he has had intermittent right testicular pain, which has been constant and increasingly painful over the past six months.

Further review of past medical history was non-pertinent. His vitals were stable upon presentation, and the physical examination showed that the superior portion of the right testicle was soft, and the inferior part was firm. Laboratory tests, including urinalysis, microscopic urinalysis, and culture, were reassuring. Doppler ultrasound demonstrated significant heterogeneity throughout the testicle, more prominent in the inferior aspect, with less pronounced Doppler flow in the right testicle.

Doppler ultrasound showed a normal size for the right testicle (4.6 x 2.0 x 3.3 cm) compared to the left testicle (4.3 x 2.0 x 3.3 cm). The right testicle was diffusely heterogeneous in echotexture, with diminished echogenicity in the mid and lower portions. No lesions were present. The right epididymis was larger than the left, with a varicocele of the pampiniform plexus that enlarged to 3 mm on Valsalva. These results, along with Doppler findings, were significant for diminished vascular supply to the right testicle (Figure 1).

**Figure 1:**
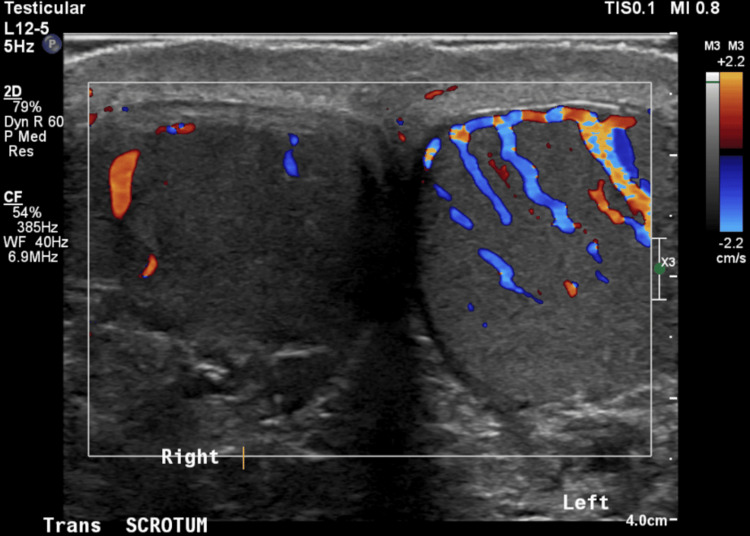
Ultrasound pelvis with Doppler Demonstrating diminished echogenicity of the mid and lower portions of the right testicle.

Although intermittent testicular torsion could not be ruled out, due to the chronicity of pain, malignancy was suspected. Following the Doppler, a urine drug screen was performed. Tumor markers (alpha-fetoprotein (AFP) and human chorionic gonadotropin (HCG)) were within normal limits.

MRI of the pelvis with and without contrast, performed two days after Doppler ultrasound, supported the absence of intra- and extratesticular masses in both testicles. MRI showed asymmetric thickening of the right epididymis compared to the left. There was questionable hypoenhancement of the right testicle compared to the left, with an absent diffusion signal in the right testicle, suspicious for testicular infarction (Figure 2). As Doppler ultrasound demonstrated an infiltrative heterogeneous appearance of the right testicle, a follow-up ultrasound was recommended in three months.

**Figure 2:**
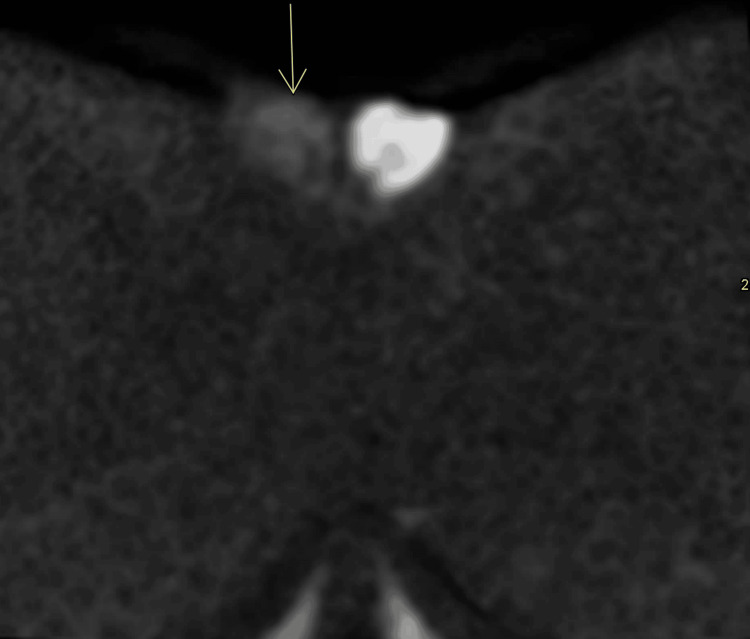
MRI pelvis Demonstrating absent diffusion signal, with asymmetric thickening and hypoenhancement of the right epididymis compared with the left.

A urology consultation and MRI findings supported a diagnosis of possible infarction. The patient was discharged home in stable condition with instructions for outpatient follow-up with the urology clinic, repeat scrotal ultrasound in three months, and use nonsteroidal anti-inflammatory drugs (NSAIDs) for pain control/inflammation. Despite multiple attempts to contact the patient by telephone and through the electronic medical record portal, the patient did not return for scheduled follow-up appointments and was subsequently lost to follow-up.

## Discussion

While typical STI present with an acute onset, clinical variation, such as chronic or cyclical onset, even in previously healthy individuals [10-12], is an important indicator for attending physicians. This is similarly reinforced by the chronic presentation of this case report.

Treatment and complications

Initial lab testing and imaging ruled out malignancy or hematologic complications secondary to an insidious stroke. The timeline of events regarding recent substance use and health history suggests STI due to these factors. Reported treatment options for STI are largely conservative and focus on antimicrobial usage, immunosuppressants such as steroids, and vitamin C supplementation for antioxidant neutralization [13]. Surveillance of sex hormone levels, such as FSH, LH, testosterone, and inhibin B, should be monitored at initial presentation and at regular intervals following conservative treatment. Surgery or laparoscopic procedures are indicated for presentations such as testicular torsion or suspected malignancy, but, to our knowledge, have not been used for STI.

Polysubstance use

STI associated with polysubstance use may be a potential etiology of the disease mechanism, a noticeable gap in the existing literature. Previous reports have discussed the possible relation between the recent use of substances, such as cannabis, methamphetamines, and cocaine, in patients presenting with normal physical exam findings, yet demonstrating signs such as perspiration, elevated temperature, and acute distress, which may be indicative of STI in a biological adult male [14-16]. Vasoconstrictive effects of substances, such as cocaine or heroin, may result in acute vasculitides or hypercoagulability in areas with less collateral circulation, such as the upper pole of the testes, making them more susceptible to infarction [17].

This case represents one of the first reported associations between STI and heroin-related polysubstance use. Previous reports describing drug-associated ischemic injury of the male genitalia have largely involved gangrenous processes rather than focal testicular infarction. Chen et al. reported scrotal gangrene following crack cocaine use, attributing the injury to cocaine-induced vasculitis and vascular degeneration [18]; however, the pathology involved the scrotal soft tissues rather than a localized infarction of the testicular parenchyma itself. Similarly, Somers et al. described cases of scrotal and penile gangrene in heroin users, thought to result from arterial embolization of particulate matter following injection drug use, as well as Fournier's gangrene after direct intrascrotal injection [19]. While these cases demonstrate the potential for substance use to cause severe genital ischemia, they do not describe STI. The closest parallel in the literature is the report by Khokhar et al. of renal infarction associated with heroin and amphetamine abuse, which highlighted proposed mechanisms including drug-induced vasculopathy, vasculitis, and activation of the coagulation cascade [20]. Our case expands this spectrum of drug-related ischemic complications by suggesting that similar pathophysiologic mechanisms may contribute to focal testicular ischemia and infarction. Given the rarity of STI and the absence of previously reported cases linked to heroin-associated polysubstance use, this case adds important evidence to the limited literature on substance-induced vascular injury affecting the genitourinary system.

## Conclusions

This case study reports a rare occurrence of STI in the setting of heroin and polysubstance use. While the etiology of STI remains incompletely understood, the vascular effects associated with substance use may represent a potential contributing factor in susceptible individuals, particularly in regions of the testis with limited collateral blood supply.
